# The predictive outfielder: a critical test across gravities

**DOI:** 10.1098/rsos.241291

**Published:** 2025-02-19

**Authors:** Borja Aguado, Joan López-Moliner

**Affiliations:** ^1^ Vision and Control of Action (VISCA) Group, Department of Cognition, Development and Psychology of Education, Institut de Neurociències, Universitat de Barcelona, Barcelona, Catalonia, Spain; ^2^ Sensorimotor Control and Learning group, Centre for Cognitive Science, Department of Human Sciences, Institute for Psychology / Centre for Cognitive Science, Technische Universitat Darmstadt, Darmstadt, Germany; ^3^ GRAD Atenció a la Diversitat, Psychology Department Faculty of Education, Translation, Sports and Psychology, Universitat de Vic - Universitat Central de Catalunya, Vic, Spain

**Keywords:** outfielder problem, optic flow, interception

## Abstract

Intercepting moving targets is a widespread challenge across many species. In humans, heuristics that use optic variables have excelled in guiding interception, relying on a closed-loop system to couple optic variables directly with direction of locomotion. This contrasts with models that explicitly recover final positions from initial trajectory conditions. However, comparing these different approaches using empirical data is challenging, as they often predict similar locomotion trajectories. We present a model based on optic variables that continuously updates predictions on the landing position in the three-dimensional scene and remaining flight time based on the outfielder’s real-time movements. A distinct feature is the model’s adaptability to different gravitational accelerations, making its predictions inherently tailored to specific environments. By actively integrating gravity, our model produces trajectory predictions that can be validated against actual paths. To compare our model with previous ones, we conducted experiments within virtual reality, strategically varying simulated gravity and physical size. The variation in gravity resulted in qualitatively distinct predictions between heuristics based solely on optic variables and our model, which incorporates gravity. The empirical trajectories, kinematic patterns and timing responses aligned well with our model’s predictions, emphasizing the importance of including environmental constants.

## 1. Introduction

**Figure 1.**
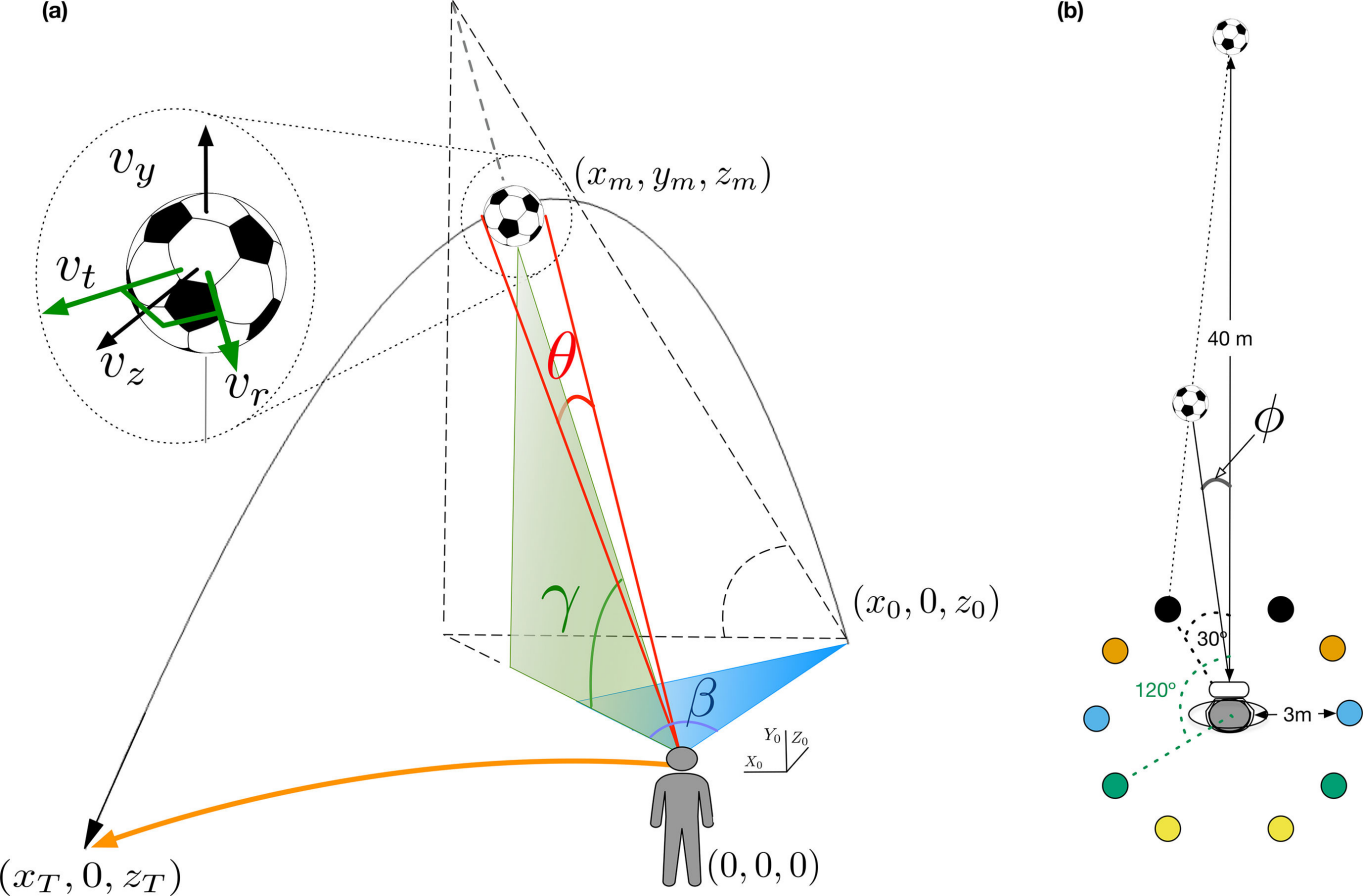
Main variables and trajectories: (a) Parabolic flight of a target and optic information available to an observer. The initial and final target positions are $\left(x_{0},0,z_{0}\right)$ and $\left(x_{T},0,z_{T}\right)$ always relative to the outfielder (e.g. *z*_0_ is the initial depth distance to the outfielder). The axes (*X*_0_, *Y*_0_, *Z*_0_) illustrate the initial frame of reference at *t* = 0 when the outfielder looks at the ball’s initial position. Note that the origin is assumed to be at eye height. The figure represents key optic variables available to the observer: elevation angle (γ), retinal target size (θ) , azimuth (β) and bearing angle (ϕ ; in panel (*b*)). 𝜓 denotes the projection of the optic trajectory, where tan𝜓 = tan 𝛾/tan 𝛽. For further definitions of these optic variables, see the model in §2. For the sake of illustration, we represent these optic variables as perceived by the outfielder at time *m* from her initial position (0, 0, 0). The inset shows different velocity vectors for the movement of the ball. The radial component 𝑣_𝑟_ is the component in the direction of the observer, which is orthogonal to the tangential component 𝑣_𝑡_ (shown in green). The vertical component 𝑣_𝑦_ and the depth component 𝑣_𝑧_ are shown in black. The LOT strategy proposes that outfielders keep 𝜓 changing linearly to control the ball. The GOAC proposes that the acceleration of tan 𝛾 is nullified and the speed of tan 𝜙 should be nearly constant to control in-depth and lateral displacement respectively. (b) Different trajectories were used in our experiment (top view). The initial position of the observer is at the origin (0, 0, 0) and the initial position of the ball was set 40 m away from the participant (*x*_0_ = 0, *y*_0_ = 0, *z*_0_ = 40). Coloured circles denote the final landing positions, 3 m from the observer, at 30°, 60°, 90°, 120° and 150° relative to the observer’s initial position (only two angle values are shown). The ball’s initial position was set 40 m away from the participant.

Consider how effortlessly an outfielder runs to catch a flyball. Explaining how this is achieved is the first step in addressing the general problem of interception, known as the outfielder problem [1–4]. The prevailing view is that the optic information gathered through our senses is sufficient to guide an outfielder’s movements to catch a flying ball [5]. This perspective has led to two primary heuristic strategies for explaining catching behaviour: (i) maintaining a linear optical trajectory (LOT) [6], where outfielders adjust their path to keep the ball’s image moving in a straight line on their retina; and (ii) cancelling optic acceleration (OAC) [1,3], which involves adjusting the outfielder’s speed to cancel the optical acceleration of the tangent of the elevation angle of the ball, primarily explaining control of the ball’s in-depth movement. The generalized optical acceleration cancellation (GOAC) strategy further proposes maintaining a constant bearing angle to control the ball’s horizontal motion [4,7,8]. For a representation of the relevant optic variables involved in the LOT, OAC and GOAC strategies, see figure 1a. While these heuristics may provide effective control, they do not account for predicting future trajectory states, which is essential for managing delays in motor planning. Instead, they handle delays through a closed-loop control system [9].

Another approach suggests that anticipating ball trajectories based on initial metric conditions provides a basis for prediction controlling behaviour [10]. However, this perspective has faced scepticism [11] owing to the complexity of the internal models required to predict projectile motion accurately [6,8,12]. Despite such scepticism, optic variables can eventually specify many properties of the trajectory, such as initial distance [13], landing position [2] and time-to-contact (TTC; [14,15]), enabling anticipatory strategies. Predictive ability is particularly evident in sports such as cricket [16], where players’ anticipatory eye movements are adjusted dynamically for varying conditions [17], highlighting a seamless integration of prediction with online visual input, suggesting an ongoing refinement of predictions [18].

Despite its importance for anticipating trajectories, Earth’s gravitational acceleration, which Gibson [19] and others in the direct-perception tradition [2] recognize as a key environmental reference, is still not explicitly considered in these models. This omission somehow overlooks the substantial influence gravity exerts on the trajectory of optic variables, reflecting a gap in how existing models account for environmental constants.

Here, we demonstrate that by integrating optic variables with known constants such as gravity and physical size of the ball, we can accurately predict when and where a ball will land relative to an actor. Furthermore, the same model can be used to update these predictions to guide the outfielder to the landing position by integrating the ongoing predictions into a simple controller. In our experiment, we used immersive virtual reality (VR; see figure 1 and §2) to manipulate ball size and gravitational acceleration, introducing values that deviated from the expected ones. Assuming that people use Earth’s gravity as a prior [20], the model predicts specific paths for the different simulated gravity values, which are qualitatively distinguishable from paths predicted by previous models using optic variables alone in the same conditions. The proposed model can predict the empirical trajectories and provide a very good account of the observed actor’s kinematics, an aspect that some previous models [21] have failed to predict.

## 2. Methods

### 2.1. Participants

We tested 12 participants (six self-identified women and six self-identified men). One participant had to be discarded owing to a particularly noisy eye-tracker’s data (the filtering procedure removed more than 10% of the trials). Participants’ ages were between 22 and 33 years with normal or corrected-to-normal vision. All the participants were naïve to the experimental goals and volunteered to participate in the experiment. This study is part of an ongoing research programme approved by the local ethics committee of the University of Barcelona in accordance with the Code of Ethics of the World Medical Association (Declaration of Helsinki).

### 2.2. Apparatus

Participants wore a head-mounted display and held a controller with their dominant hand (all were right-handed). The experiment was performed on an Intel i7-based PC (Intel, Santa Clara, CA, USA)(i7-9700F). The stimuli were rendered using an NVIDIA GeForce (RTX 2060 SUPER) and sent to a wireless HTC Vive Pro head-mounted display (HMD) at 90 Hz per eye. The position (*x*, *y*, *z*) in the virtual space of the HMD as well as the rotation angles (yaw, pitch, roll) were tracked at 90 Hz by four SteamVR Base Stations (2.0), positioned 7.8 m × 4.1 m apart and mounted approximately 2.25 m above the floor. These 6 degrees of freedom data points allowed us to fully specify the participant’s position with respect to the ball. Eye movements were recorded using a built-in eye tracker (Tobii Technology, 2011) at 90 Hz.

### 2.3. Stimuli

We used 10 different trajectory angles (figure 1b) in which the ball followed a parabolic trajectory towards the observer. The ball’s initial position (*x* = 0, *z* = 40) was 40 m from the observer and laterally aligned with the observer’s initial position (lateral *x* = 0, depth *z* = 0). The interception location was always located 3 m away from the observer’s starting position describing horizontal trajectory angles of ±30°,60°,90°,120°,150° (negative denotes left side and 90° corresponds to *z* = 0) with respect to the observer’s initial location (figure 1b). We used both the Earth gravitational acceleration (9.81 m s^−2^) at sea level and soccer ball size (0.22 m diameter) ± 10% of their respective standard values. In total, we had 90 (10 trajectories × 3 gravities × 3 sizes) conditions.

The ball’s initial height was vertically aligned to eye height on a trial-by-trial basis to account for any HMD slip and postural changes. Flight duration (or TTC) was randomly selected from a uniform distribution ranging from 3.15 to 3.85 s ( ± 10% of 3.5 s). The initial and final positions, flight duration and gravitational acceleration fully determine the initial vertical and horizontal velocities of the ball. Air resistance and other complex effects were neglected.

### 2.4. Procedure

Prior to the experimental procedure, the participant and the experimenter tossed a standard-sized soccer ball (diameter 22 cm) back and forth to develop familiarity with the ball’s size. Each participant underwent a total of 10 blocks of 90 trials each. Each block was presented with one repetition of all combinations of gravity, size and trajectory. The task was self-paced and each block lasted for about 10−16 min. Participants completed 20 training trials before the main experimental procedure to familiarize themselves with the task and VR environment. The eye tracker was calibrated before each block. Calibration accuracy was tested with a custom programme and always remained below 1.89° error. Each trial was conducted as follows: (i) the participant was instructed to align both body and gaze while looking at the ball. Once aligned, the participant launched the ball by pressing a button; (ii) once the ball was in the air, the observers followed the ball visually while moving towards the interception point; and (iii) after completing 90% of the flight time, the ball was occluded and was no longer visible. The participants were instructed to head towards the position where they thought the ball would land and press a button to estimate the TTC (when the ball was again at eye height). Participants did not receive any feedback on their performance.

### 2.5. Data analysis

Gaze was categorized as being on the ball if the absolute vertical distance between the ball and gaze was lower than 6.5° [22]. The probability of gaze being on the ball was, on average, larger than 90% during the entire time the ball remained visible (see the electronic supplementary material, figure S1). As the direction of the ball (left or right) did not affect the temporal errors committed by our participants (*t*_10_ = −0.05, *p* = 0.961) and our observers’ average heading angle (*t*_10_ = −0.953, *p* = 0.363), we combined the results for right- and left-handed trajectories for further analysis by rotating leftward locomotion trajectories to align with right-handed parabolic trajectories. For the final analysis, we removed those trials in which the frame rate was inconsistent, that is, the mean frame rate was lower than 81 frames/s. In addition, we removed trials in which the eye was detected by the eye tracker in less than 90% of the flight time and trials where the participant did not look at the ball at all (absolute average vertical distance between the ball and gaze was larger than 15°). Finally, we excluded trials in which the response time was longer than 5 s. This procedure eliminated 265 trials (2.67% of the total).

### 2.6. Locomotion

To analyse the paths travelled across trajectories, gravities and sizes, we first normalized the paths based on the percentage of the distance covered. Each trial was then divided into 100 steps, with the 100th step corresponding to the moment the participant pressed the trigger, indicating that the ball returned to eye height. We then computed the average heading angle for each participant, trajectory, gravitation, ball size and step number. The heading is defined as the angle between the participant’s movement direction and the initial position of the ball (0° indicates movement directly along the line connecting the participant’s starting position to the ball’s starting position). For the ANOVA analysis, we aggregated the heading data by subject, trajectory, gravity and ball size, focusing on the steps before occlusion and once the actor began moving. Prior to the analysis, we visually inspected the densities to check for a normal distribution of the angles. In this analysis, participants were treated as a random effect, while trajectory, gravity and ball size were treated as fixed effects.

### 2.7. The model

Figure 1a illustrates the general case of parabolic movement, with the observer’s initial position at the origin of the coordinate system, given by $x_{0}^{o},y_{0}^{o},z_{0}^{o}=\left(0,0,0\right)$. Assume that the observer’s eye height is at plane y=0, and at time t=0, a ball is launched from the point $\left(x_{0},0,z_{0}\right)$ at eye height. The ball is shown at time t=m and returns to eye height at time t=T, located at point $\left(x_{T},0,z_{T}\right)$.

We introduce the model in stages. To simplify the derivation, we begin by presenting the model’s perceptual estimates within a fixed reference frame centred at the observer’s starting position, as illustrated in figure 1a. This approach provides accurate perceptual estimates without assuming any specific observer movement, which would complicate the derivation. We will later demonstrate empirically that these predictions generalize across different observer positions relative to the ball’s landing position. By establishing the model’s accuracy across various observer positions, we build a foundation for extending it to an egocentric frame that moves and rotates with the observer. This extension will allow perceptual estimates to integrate directly with the movement controller presented later.

### 2.8. Previous definitions

The following terms are defined:

— s, physical diameter of the target;
— $\left(x_{m},y_{m},z_{m}\right)$, position of the ball at time m;
— $v_{0y}$, initial vertical component of the ball motion;
— $v_{y},v_{x},v_{z}$, vertical, lateral and depth components of parabolic motion (see figure 1a);
— $v_{r}$, radial component of the parabolic motion from the observer point of view (see figure 1a);
— $v_{t}$, tangential component of the parabolic motion from the perspective of the observer (normal to the radial component);
— d, distance of the ball to the observer;
— T, the total flight time (i.e. ball is above eye height) and is given by: $\frac{2v_{0y}}{g}$;
— γ, vertical angle with respect to the observer’s eye height (elevation angle);
— θ, angle subtended by the ball on the retina (retinal size);
— β, horizontal angle between the projection of the ball on the floor, observer and initial target position (azimuth, see figure 1a);
— ϕ, horizontal angle between observer’s gaze and target (bearing angle, see figure 1b); and
— $T_{c}$, remaining time for the target to return at eye height after movement onset at some specific position or moment of time.

Target position (e.g. x, z, y), angular variables (e.g. θ, γ, β), their temporal derivatives and remaining $T_{c}$ are time dependent variables (e.g. $z_{t}$), but we will drop time indexes for simplicity. The height y of the ball at time t is given by (using $y_{0}=0$):


(2.1) $$y=v_{0y}t-\frac{gt^{2}}{2}.$$

We will further assume that the depth position (z) of the ball is given by:


(2.2) $$z=\frac{s}{\theta }cos\left(\gamma \right).$$

The tangent of the elevation angle γ at time t can be derived by substituting the total flight time into the vertical position (2.1):


$$A=tan\left(\gamma \right)=\frac{y}{z}=\frac{v_{0y}t-\frac{gt^{2}}{2}}{z}.$$

We can rewrite $v_{0y}t-\frac{gt^{2}}{2}$ in terms of T by noting that $T=\frac{2v_{0y}}{g}$, which implies $v_{0y}=\frac{gT}{2}$. Substituting this back, we get


(2.3) $$A=tan\left(\gamma \right)=\frac{\frac{gT}{2}t-\frac{gt^{2}}{2}}{z}=\frac{\frac{gT}{2}\left(T-t\right)}{z}=\frac{1}{2}gt\frac{T_{c}}{z}.$$

Therefore, when t≠0,


(2.4) $$T_{c}=\frac{2Az}{gt},$$

where $T_{c}=T-t$ is the remaining TTC (i.e. the ball returns at eye height) at time t.

### 2.9. Predictive estimation of $T_{c}$, $x_{T}$ and $z_{T}$ when t=0

In this case, when Δt tends to 0, we can arrive at


(2.5) $$\overset{˙}{\gamma }\Delta t=v_{0y}\Delta t-v_{z}\Delta t\Delta \gamma =v_{0y}\Delta t+o\left(\Delta t\right),$$

where o is the little o of Landau, so we can have


(2.6) $$v_{0y}=\overset{˙}{\gamma }z.$$

Therefore,


(2.7) $$T_{c}=\frac{2v_{0y}}{g}=\frac{2\overset{˙}{\gamma }z}{g}=\frac{2s\overset{˙}{\gamma }}{g\theta }.$$

Equation (2.7) defines the remaining flight time ($T_{c}$) as a function of optic variables, gravitational acceleration g and physical size s. This equation corresponds to the case when the ball falls on the initial location of the observer [14]. Note that $T_{c}$ is time-dependent and signals the remaining time at t=0 irrespective of the observer’s position (see the electronic supplementary material, figure S6 for temporal errors associated with the use of equation (2.7) at different observer’s positions at trajectory times).

With respect to $z_{T}$, we have that


(2.8) $$z_{T}=z-v_{z}T_{c}=\frac{s}{\theta }cos\gamma -\left(v_{t}sin\gamma +v_{r}cos\gamma \right)T_{c}.$$

We have decomposed the velocity component in depth $v_{z}$ into the tangent $v_{t}$ and radial component $v_{r}$ (see figure 1a). Since $v_{t}=\dot{\gamma }\frac{s}{\theta }$ and $v_{r}=s\frac{\dot{\theta }}{\theta^{2}}$, we can obtain $z_{T}$ once we know $T_{c}$:


(2.9) $$z_{T}=\frac{s}{\theta }cos\gamma -\left(\overset{˙}{\gamma }\frac{s}{\theta }sin\gamma +s\frac{\overset{˙}{\theta }}{\theta^{2}}cos\gamma \right)T_{c}.$$

The final position in depth is specified at the initial moment (t=0) by optic variables and constants g and s. This formulation, as is often the case in real scenarios, provides accurate spatial predictions once the outfielder is looking at the ball, which corresponds to the experimental condition at *t =* 0 (see figure 1b), so that the depth axis in the initial fixed frame of reference aligns with the outfielder’s line of sight. The limiting factor in estimating $z_{T}$ is the rate of expansion ($\dot{\theta }$). In our simulations, we introduced noise values for $\dot{\theta }$ that exceeded known reported thresholds of 11% [23]. Despite this, estimates of $z_{T}$ remained robust. This robustness can be attributed not only to the dependence on $v_{r}$ (which is influenced by $\dot{\theta }$) but also to the contribution from $v_{t}$.

In relation to $v_{x}$, like before, it is easy to solve it (see figure 1a):


(2.10) $$v_{x}=\overset{˙}{\beta }z=\overset{˙}{\beta }\frac{s}{\theta },$$

therefore,


(2.11) $$x_{T}=v_{x}T_{c}=\overset{˙}{\beta }\frac{s}{\theta }T_{c}=\frac{2}{g}\frac{s^{2}}{\theta^{2}}\overset{˙}{\gamma \overset{˙}{\beta }}.$$

The final lateral position is fully specified by optic variables together with g and s at t=0.

### 2.10. Estimation of $T_{c}$, and $x_{T}$ when $z_{T}=0$

In this scenario, the ball's final position is at the same depth as the initial position of the observer $z_{T}=0$. Since, during the trajectory, z>0, $v_{z}<0$ and $z_{T}=0$, according to equation (2.3), we have that the tangent of γ is


$$A=-\frac{gt}{2v_{z}}.$$

Therefore,


$$\overset{˙}{A}=-\frac{g}{2v_{z}}.$$

Furthermore, when the target is at position z, the remaining time is $T_{c}=\frac{z_{T}-z}{v_{z}}$, and since $z_{T}=0$ we have


(2.12) $$T_{c}=\frac{-z}{v_{z}}=\frac{2\overset{˙}{A}z}{g}=\frac{2s\overset{˙}{A}cos\left(\gamma \right)}{g\theta }=\frac{2s\overset{˙}{\gamma }}{g\theta cos\left(\gamma \right)}.$$

Since cosγ at t=0 is 1, equations (2.7) and (2.12) are equivalent.

As for $x_{T}$ (i.e. the final lateral position), we can calculate it using the formula:


(2.13) $$x_{T}=v_{x}T_{c}=\overset{˙}{\beta }\frac{s}{\theta }cos\gamma T_{c}.$$

Substituting $T_{c}$ in equation (2.13) with equation (2.12) we finally have:


(2.14) $$x_{T}=\frac{2}{g}\frac{s^{2}}{\theta^{2}}\overset{˙}{\gamma }\overset{˙}{\beta },$$

we obtain the same expression as in equation (2.11).

#### 2.10.1. Adaptation of model-based estimates to observer movement

**Figure 2.**
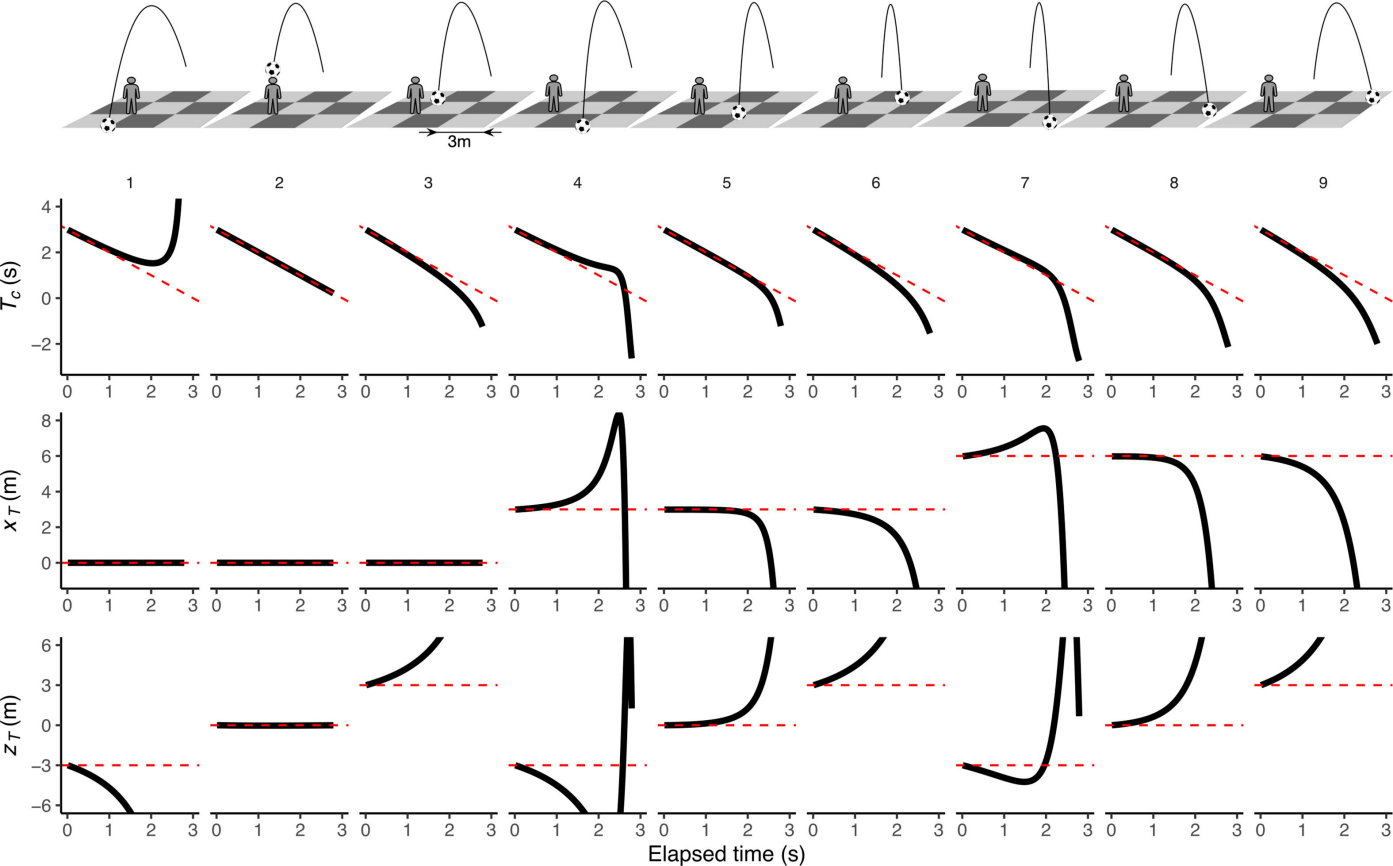
Model-based predictions of TTC (𝑇_𝑐_), lateral position (𝑥_𝑇_ ) and depth position (𝑧_𝑇_ ) across different final positions of the ball relative to a static observer: the top row illustrates various scenarios where the ball lands at different relative positions with respect to the (static) observer on a floor of 9 m × 9 m. The simulated ball is located 40 m from the observer with an initial time to contact (𝑇) of 3 s. This fixed time is used for illustration purposes, although the predictions are invariant to both initial TTC and initial distance. The graphs below show the corresponding model estimates over time: the first row of results presents the temporal estimate (𝑇_𝑐_), the second row shows the estimated final lateral position (𝑥_𝑇_) and the third row displays the estimated final depth position (𝑧_𝑇_ ). Solid black lines indicate model estimates, while dashed red lines represent the actual values.

In the previous derivations, we obtained perceptual estimates for the remaining TTC ($T_{c}$) and the final lateral and depth positions relative to a static observer in two specific cases: at the initial time (t=0) and when the ball lands at the same depth as the observer in the initial frame of reference. Notably, the initial estimates (t=0) provide accurate values for $T_{c}$, $x_{T}$ and $z_{T}$, offering valuable predictive information, as illustrated in figure 2. The model’s accuracy varies over time depending on the ball’s trajectory. For instance, the output remains accurate throughout the trajectory when the ball lands precisely at the observer’s position (column 2 in figure 2). Accuracy is also maintained for a longer duration when the ball lands at the same depth as the observer (compare columns 8 and 9 in figure 2) and when it lands laterally closer to the observer (compare columns 5 and 8 or 9 in figure 2).

The model’s ability to generalize across different observer positions, coupled with the initial accuracy of estimates regardless of the observer’s location, allows us to extend the model equations to scenarios involving observer movement. As the observer moves, the accuracy of these estimates will depend on both the observer’s movement and the timing of those movements. As introduced in the controller below, the same equations—equations (2.12) (for $T_{c}$), (2.14) (for $x_{T}$) and (2.9) (for $z_{T}$)—can be dynamically updated by substituting the angular variables as they change in response to the observer’s motion. This updating process illustrates how the model can maintain accuracy through incremental adjustments depending on the observer’s movements. In other words, although the core equations are derived in a static reference frame, they can be applied in a dynamic context (see the electronic supplementary material, figure S6) and thereby integrate seamlessly with the observer’s movement to provide reliable perceptual estimates that guide locomotion.

#### 2.10.2. Controller dynamics

The dynamics of the controller were inspired by the controller put forward in [8]. The radial and tangential accelerations are controlled respectively as follows:


(2.15) $$\overset{˙}{v_{r}}=\zeta_{r}*v_{r}+tanh\left(\left(z_{T}\text{/}T_{c}\right)*c_{r}\right)*k_{r},$$

(2.16) $$\overset{˙}{v_{t}}=\zeta_{t}*v_{t}+tanh\left(\left(x_{T}\text{/}T_{c}\right)*c_{t}\right)*k_{t},$$

where $\zeta_{r}$ and $\zeta_{t}$ are damping terms and, $k_{r}$ and $k_{t}$ are stiffness terms, and $c_{r}$ and $c_{t}$ are thresholds terms that approximate a sigmoidal response to the control estimates. The values of ζ, k and c were obtained with an optimization procedure using the *optim* function of the R software. The optimization minimized the negative log likelihood (*nll*) between the model predictions and the averaged (*x*, *z*) data points in our dataset. The optimization procedure was applied to trajectories in the 1G condition only. The values ${\dot{v}}_{r}$ and ${\dot{v}}_{t}$ were integrated at each frame to update both velocity components of the actor (see R code in file ‘2_Controller.qmd’; Δt = 0.05 s.). The resulting fitted parameters were: $\zeta_{r}$ = 0.735; $k_{r}$ = 1.032; $c_{r}$ = −0.565; $\zeta_{t}$ = 0.494; $k_{t}$ = 1.545; $c_{t}$ = −0.698.

After this optimization procedure, we run another optimization procedure minimizing the *nll* to obtain the weight of the gravitation prior to employing averaged data across the three gravitations and the five possible trajectory angles. The fitted value corresponded with a prior weight of 0.192 (w=0.192).

Additionally, we implemented a second heuristic controller to fit our data. In this case, the simulated outfielder’s movements were guided by linking them to the optic variables used in Fink’s [8] approach that implements the GOAC strategy. Like their implementation, the jerk in the radial and tangential components of the movement relied on the first and second derivatives over time of tanϕ and tanγ, respectively:


(2.17) $$\overset{˙}{v_{r}}=\zeta_{r}*v_{r}+tanh\left(d^{2}tan\left(\gamma \right)\text{/}dt^{2}*c_{r}\right)*k_{r},$$

(2.18) $$\overset{˙}{v_{t}}=\zeta_{t}*v_{t}+tanh\left(dtan\left(\phi \right)\text{/}dt*c_{t}\right)*k_{t}.$$

Similarly, $\zeta_{r}$ and $\zeta_{t}$ are damping terms and $k_{r}$ and $k_{t}$ are stiffness terms; $c_{r}$ and $c_{t}$ are thresholds terms that approximate a sigmoidal response to the control estimates. The values of ζ, k and c were obtained as before by minimizing the *nll* between simulated model trajectories and averaged (*x*, *z*) data points in our dataset. The values ${\ddot{v}}_{r}$ and ${\ddot{v}}_{t}$ were integrated at each frame to update both actor velocity components (see R code in file ‘2_Controller.qmd’). We initially employed this procedure to estimate the values of $\zeta_{r}$, $\zeta_{t}$, $k_{r}$, $k_{t}$, $c_{r}$ and $c_{t}$ in the 1G condition with the resulting fitted parameters: $\zeta_{r}$ = 5.907; $k_{r}$ = 2.797; $c_{r}$ = 39.592.; $\zeta_{t}$ = 7.289; $k_{t}$ = 13.998; $c_{t}$ = 17.134. Subsequently, we applied this set of parameters to test the model’s performance in the gravity conditions other than 1G. However, since the predictive model incorporates an additional parameter (w) when tested in gravities different than 1G, we conducted further fitting procedures, as described in the electronic supplementary material, figures S4 and S5, to enhance the flexibility of the heuristic model. These additional fitting procedures involved adjusting different controller terms in conditions other than 1G to achieve a better fit to the data.

## 3. Results

### 3.1. Gravity affects spatial trajectories

**Figure 3.**
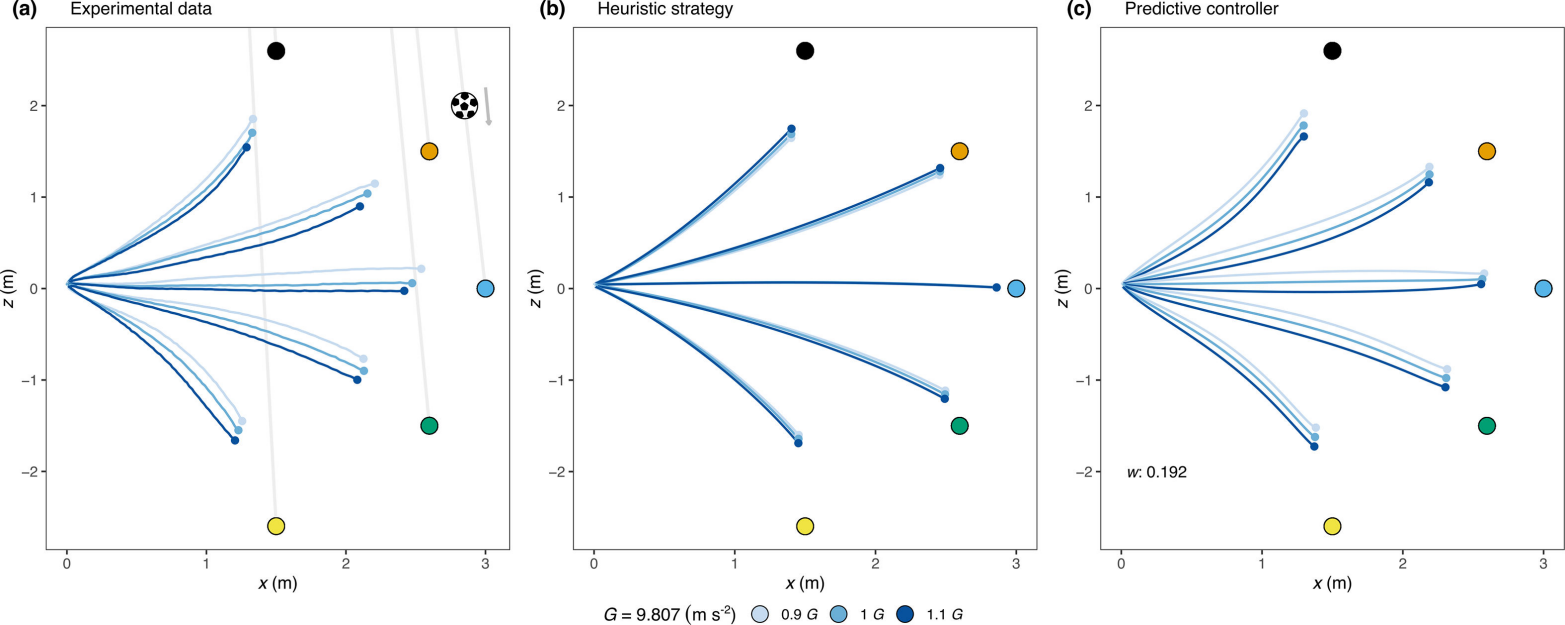
Empirical and simulated paths: (a) Average empirical paths followed by participants in the experiment. Data were first averaged across trajectory angles for each observer and time-step (see §2.6), and then a mean trajectory across observers was calculated. The final 200 ms of flight were excluded to avoid averaging over a small number of trials owing to variations in response times. Grey lines indicate the trajectories followed by the ball. (b) Paths simulated using a controller guided by GOAC heuristic strategy. (c) Paths simulated using a controller guided by a predictive strategy. See §2.10.2 for details on the trajectory predictions for both strategies. The weight (*w*) of Earth’s gravitational acceleration (𝑔_𝑒_) that provided the best fit, when combined with simulated gravitational acceleration (𝑔_𝑠_) as in 𝑔 = 𝑤𝑔_𝑒_ + (1 − 𝑤)𝑔_𝑠_, was *w* = 0.192.

Figure 3a shows the average direction of the spatial trajectory across participants for the different simulated gravities. The electronic supplementary material, figure S1 shows individual paths. In agreement with previous studies [6,8], participants did not follow a linear path. Instead, when the ball landed in front of them, they followed a slightly convex trajectory, and when the ball landed behind them, their path was concave. Importantly, when analysing the segment of the trajectory before occlusion and during the actor’s movement, gravitation had a significant effect on heading angle (figure 3a, *F*_2,20_ = 64.42, *p* < 0.001, $\eta^{2}$ = 0.19). When gravity exceeded terrestrial levels, participants’ paths deviated more from the ball’s trajectory: becoming more convex when running forward and less concave when running backward.

To isolate gravity’s influence on initial vertical velocity ($v_{0y}$) and flight duration, we analysed their impact on trajectory variation. This analysis points to gravity as the primary factor altering trajectories, with $v_{0y}$ variations showing no correlation with landing position (electronic supplementary material, figure S2A).

Empirical data comparisons with the GOAC heuristic and our model underscore our model’s closer alignment with observed data across gravity conditions (figure 3b,c). These predictions stem from the respective controllers’ output. Figure 3b shows that while the GOAC heuristic can replicate curved paths, it fails to capture the empirical variance across different gravities as observed in front of the actor: with larger gravities producing less convex paths. Conversely, our model accurately replicates the curved paths and heading directions for different gravity values, with increased gravity resulting in more convex forward paths and reduced concavity when moving backwards. Cross-correlation analyses, which compare actual and predicted headings, further reinforce this alignment, showing higher correlations for our model (see the electronic supplementary material, figure S2B).

Our model achieves an optimal fit by differentiating between trajectories under various gravitational conditions, using a weighted combination of Earth’s gravitational acceleration ($g_{e}$) and simulated gravity ($g_{s}$), set at 0.192 and 0.808, respectively. See the electronic supplementary material, figure S3 for the simulated paths that result from different weighted combinations of Earth’s gravity with simulated gravitational accelerations. This weight reflects a strategy where participants rely partially on an Earth’s gravity acceleration. It is important to note that participants have direct optical information available to estimate the simulated acceleration $g_{s}$ through trial-by-trial changes in the following combination ($\overset{˙}{\gamma }/\theta cos\gamma$) of optic variables, which are part of the remaining TTC estimation $T_{c}$ (see equations (2.7) or (2.12)). As this computation relies on angular information alone, no further assumptions about the physical size *s* are necessary. The electronic supplementary material, inset of figure S2B shows the average output of the combination of these optical variables.

While the initial impression might suggest that our fitting procedure favours our predictive model primarily owing to the additional weighting parameter, it is essential to highlight a pivotal observation from figure 3: the symmetry observed in the heuristic controller’s predictions for different gravities, both for trajectories landing in front and behind. This symmetry emerges from the principles of the GOAC model and reflects a qualitative difference between the heuristic controller’s predictions and the empirical data. To investigate whether this qualitative discrepancy can be rectified by introducing more flexibility into the heuristic controller, we conducted additional fittings. In these fittings, we granted the heuristic controller more flexibility by freeing one controller term which consists of two parameters and subsequently, two controller terms (electronic supplementary material, figure S4, S5 and table S1). Despite this heightened flexibility, the heuristic controller could not account for the trajectory trends observed in the data. These tests demonstrate that the qualitative differences observed between the heuristic controller’s predictions and the empirical data cannot be resolved to align the predicted paths with the actual observed ones.

While the simulated gravitational acceleration had a pronounced impact, the ball size influenced trajectories only marginally (*F*_2,20_ = 2.99, *p* = 0.083, $\eta^{2}$ = 0.005). Our model predicts an effect of size, specifically if participants use the mean size as a prior owing to familiarity. Though a one-tailed test could be considered based on this prediction, the modest effect size warrants a cautious interpretation, suggesting reliance on the actual size without drawing on prior knowledge. This perspective is expanded upon in §4. There was no significant interaction between the simulated gravitational acceleration and ball size (*F*_4,40_ = 1.02, *p* = 0.402, $\eta^{2}$= 0.003).

### 3.2. Predictive model

Unlike heuristics in which the outfielder movement is coupled with the ball through an error-nulling tactic, our actor uses optic variables combined with knowledge about physical size and gravity to obtain first an estimate of the remaining flight time $T_{c}$ (equation (2.12)):


$$T_{c}=\frac{2s\overset{˙}{\gamma }}{g\theta cos\left(\gamma \right)}.$$

In this equation, s represents the ball’s physical size, and g is gravitational acceleration, as previously discussed, influenced by weights (w and 1−w) assigned to terrestrial (1G) and simulated gravitational acceleration. Both γ (elevation angle) and θ (ball’s angular size) are time-varying factors, as illustrated in figure 1a. At the trajectory’s onset (i.e. t=0), this expression simplifies to $T_{c}=2s\dot{\gamma }/g\theta$ (see equation (2.7)). It is crucial to emphasize the role of the observer’s movement in this context. The equation offers an estimate of $T_{c}$ for a specific system state. This state is dynamic and is influenced by how the observer moves. Specifically, angular variables γ and θ are directly affected by the observer’s movement. As such, the way in which participants move and adjust their position plays a crucial role in the real-time calculations and accuracy of $T_{c}$. Importantly, as illustrated in figure 2, this initial temporal estimate quickly allows the actor to obtain an accurate estimate of the final lateral position $x_{T}$ of the ball relative to her, thus making spatial and temporal information inseparable [24]:


$$x_{T}=\frac{s\overset{˙}{\beta }}{\theta }T_{c}=\frac{2}{g}\frac{s^{2}}{\theta^{2}}\overset{˙}{\gamma }\overset{˙}{\beta },$$

where $\dot{\beta }$ is the rate of change of the azimuth angle (figure 1a) and the final position $z_{T}$ in depth relative to the observer:


$$z_{T}=\frac{s}{\theta }cos\gamma -\left(\overset{˙}{\gamma }\frac{s}{\theta }sin\gamma +s\frac{\overset{˙}{\theta }}{\theta^{2}}cos\gamma \right)T_{c}.$$

**Figure 4.**
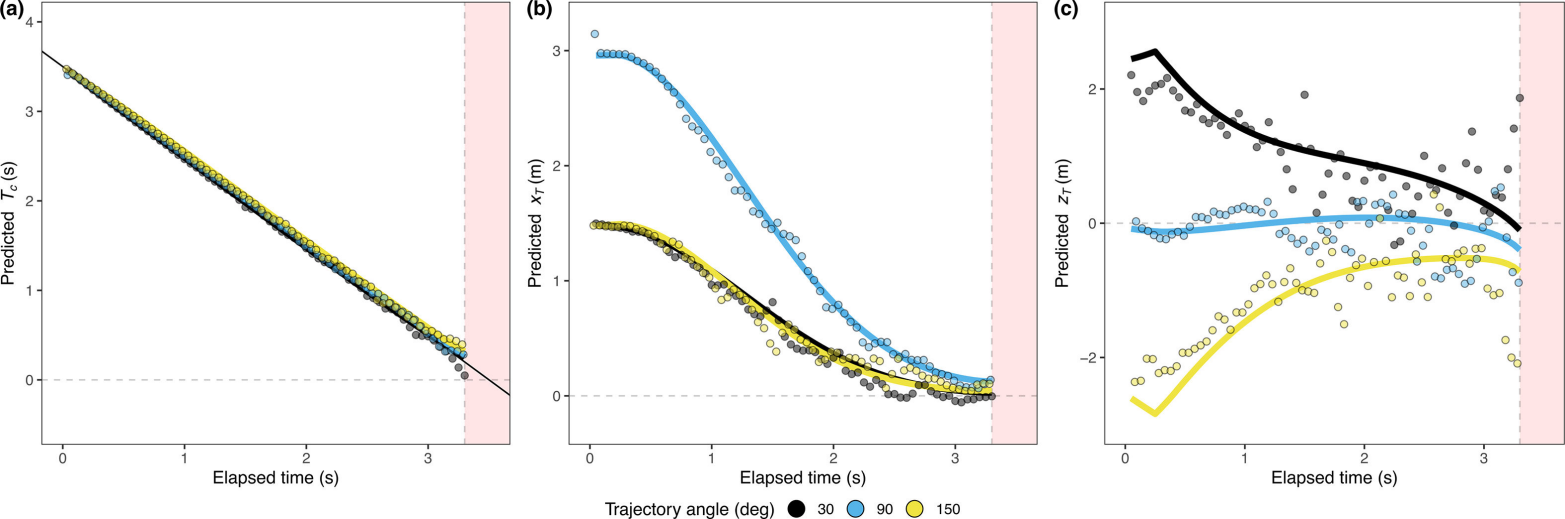
Temporal course model estimates: Panels (a), (b) and (c) show estimates based on our predictive model for successful catches: remaining TTC (𝑇_𝑐_) and distance to the ending position in the *x-* (𝑥_𝑇_) and *z*-axis (𝑧_𝑇_), respectively, relative to the observer. The estimates (equations (2.12), (2.14) and (2.9) based on the actual movements are represented by points, and the best fit of the model controller across all the trials is represented by a line. The colour code represents three of the trajectory angles tested in the experiment. The red area indicates the occlusion interval.

Unlike the previous model derivation in the methods, it is important to note that the reference frame in which $x_{T}$ and $z_{T}$ are updated to simulate the trajectories is not fixed anymore; it rotates as the observer turns. Figure 4 shows the time course of these three estimates ($T_{c},x_{T}$ and $z_{T}$ using, respectively, equations (2.12), (2.14) and (2.9) for three of the trajectory angles, considering the changing position of the actor under terrestrial acceleration. In other words, figure 4 tells us how the temporal and spatial predictions are updated. The data presented in this figure are representative of trials in which the participants were able to catch the ball. We classified a trial as a catch only if the observer was within 0.5 m of the ending point at the time of contact. We plot both estimates based on our actors’ actual movements (dots) and the resulting best fit (line) from the predictive controller (see §3.3). Notably, while the actual paths in space were curved, participants took a route where timely changing positions resulted in a linear decrease in the remaining flight time estimate, $T_{c}$, as shown in figure 4a. When an actor’s movements linearize $T_{c}$, it consistently reduces the estimated final lateral position according to the previous equations (figure 4b). Finally, figure 4c shows the corresponding estimate for the final depth position of the ball relative to that of the observer. The deviations of data points from the line in figure 4 stem from variations in actor positions at the same time frames for these trajectory angles, influencing the updated estimates. The increased fluctuation observed in figure 4c is a result of noisier estimates when updating $z_{T}$ owing to the consideration of retinal expansion of the ball. One must recall that the accuracy of these estimates depends on both the actor’s position and the timing of these positions. At the flight’s onset (figure 2), these estimates accurately reflect the relevant physical aspects in the three-dimensional environment (e.g. landing positions and flight time), regardless of the observer’s initial position relative to the ball; but accuracy of later estimates will depend on the actual movement of the actor. The model can provide sufficient information to guide the movement to obtain a linear decrease in the remaining flight time. The electronic supplementary material, figure S6 and an animated version (temporal_error_map.gif) show an error map of the temporal estimates (based on equation (2.12)) for all possible positions at different times. It can be seen that, irrespective of the position of the observer at the target launch, the error of temporal estimates is minimal at the beginning.

### 3.3. The controller

To generate the trajectories based on the spatio-temporal estimates shown in figure 4 and make the predictions plotted in figure 3c, we implemented a simple dynamical model in which an actor controls the radial velocity ($v_{r}$, movement towards the ball) and tangential speed ($v_{t}$, orthogonal component to $v_{r}$) of her movement independently (see §2.10.2). The actor was required to wait for 350 ms before initiating movement, which included a simulated processing sensorimotor delay of 200 ms. At each time, the estimates of the two velocity components were computed as $v_{r}=z_{T}/T_{c}$ and $v_{t}=x_{T}/T_{c}$, and an acceleration component was computed in the dynamical model and integrated to keep the velocity of the actor close to the estimates. Our model estimates provide the actor information of the average velocity at which she must move laterally and in depth to reach the final position of the ball, with the possibility of translating these velocities into action-scaled information [25]. This strategy appears intuitive and is consistent with the subjective feeling of quickly knowing whether one would be able to catch the ball [26] and decide to start running.

### 3.4. Movement kinematics

**Figure 5.**
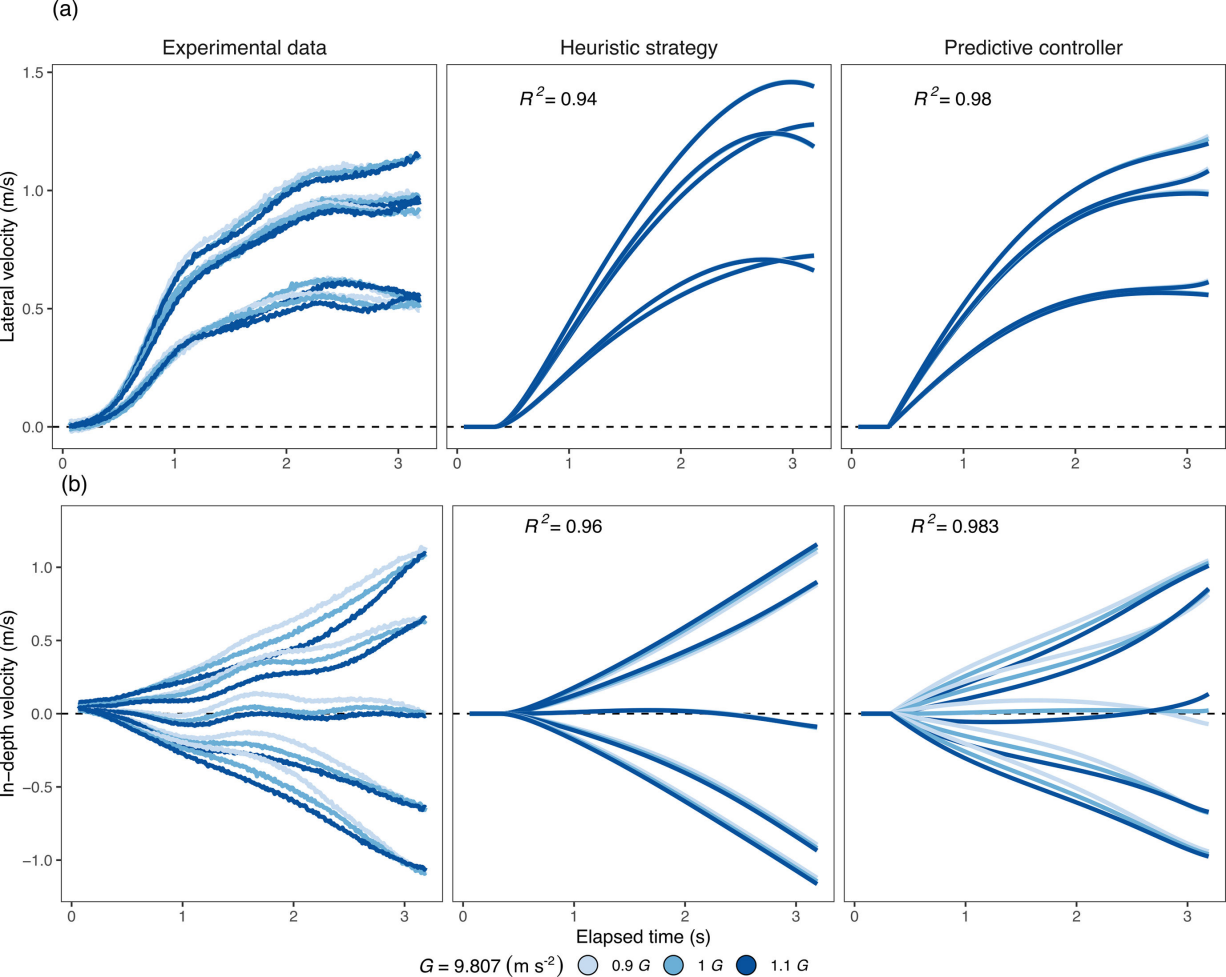
Movement kinematics: average velocity for the lateral (a) and in-depth (b) motion across trajectories and gravitations (colour coded). The columns present experimental data, heuristic predictions and predictive model predictions. Experimental data were smoothed by using a moving average (window of 0.1 s) that was run in the two directions to prevent a phase shift.

The predictive model, based on the controller, we have just presented, effectively accounts for the observed kinematics. In the first column of figure 5, we display the average lateral velocity component (figure 5a) and the depth velocity component (figure 5b) across participants, encompassing various trajectory angles and gravitational conditions. The second and third columns show the predictions for the GOAC strategy and our predictive strategy, respectively. Our predictive model exhibits a better fit to the kinematic data ($R^{2}$ = 0.98, lateral; $R^{2}$ = 0.983, in-depth) compared with the heuristic counterpart ($R^{2}$ = 0.94, lateral; $R^{2}$ = 0.96, in-depth). The GOAC model fails to capture the observed lateral deceleration during the latter portion of the trajectory, typically occurring after 1.5 s. This limitation in replicating the deceleration aligns with a known shortcoming of the LOT model too, as discussed in [21]. Importantly, our model shows some sensitivity to gravitational variations in the depth component of movement, where gravity exerts a substantial influence (*F*_2,20_ = 35.22, *p* < 0.001, $\eta^{2}$ = 0.058) considering the whole trajectory. In this dimension, we observe a significant impact of gravity on the trajectories. However, our model falls short in capturing the variations in lateral movement, where gravity still has a significant effect (*F*_2,20_ = 8.35, *p* = 0.02, $\eta^{2}$ = 0.018).

### 3.5. Final stage: temporal judgments

**Figure 6.**
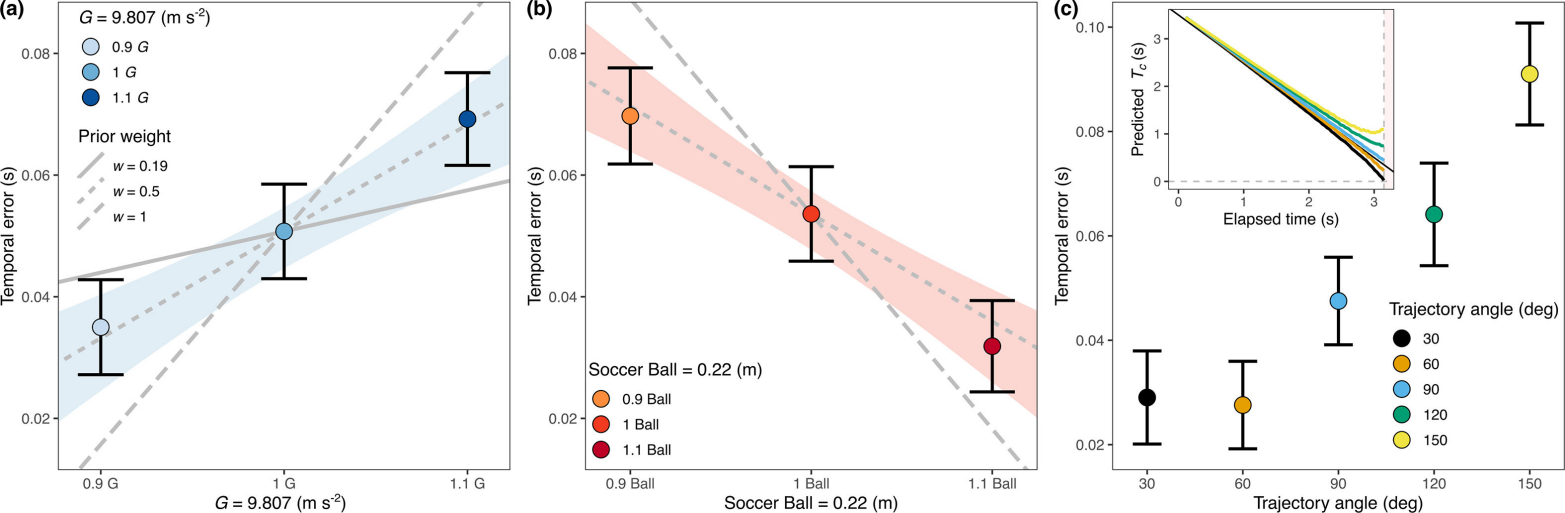
Temporal error per gravity, size and trajectory angle: averaged temporal errors committed by our participants across gravitations (a), ball sizes (b) and trajectory angles (c). Error bars indicate 95% CI. Lines in panels (a) and (b) indicate predicted temporal errors, assuming a combination of prior knowledge (*W*) and veridical physical variables (1 *w*) for gravity in panel (a) and size in panel (b). The shaded area indicates a 95% CI for the fitted linear models that only includes the slope resulting from a prior weight of a 0.5 for gravity and size. In panel (c), the inset represents the average predicted TTC per trajectory derived from our participants action.

According to our model predictions, the assumption of Earth’s gravity would lead to an overestimation of TTC when an actor is exposed to gravitations larger than terrestrial (1G). Indeed, this is the pattern we observed (figure 6a; gravity-main effect: *F*_2, 20_ = 33.802, *p* < 0.001, $\eta^{2}$ = 0.004). The slopes in figure 6a predict the trend of the expected response patterns when Earth’s gravity is given full weight (w=1) and when weaker weights are assumed (w=0.192 and w=0.5). The 95% confidence interval (CI) of the fitted slope across temporal errors does not include the weight of 0.192 across gravitations (see shaded area in figure 6a). The mean weight for Earth’s gravity that accounts for the separation of the spatial trajectories in locomotion across gravitational accelerations is smaller (*w* = 0.192) than the weight explaining the effects of gravity in the temporal estimation task (figure 6a). Note that since visual information is no longer available during the final temporal estimation phase, one would rely more heavily on prior knowledge of gravity. We resume this point in §4. The prior assumption of a soccer ball size would lead to underestimation of TTC if the ball size is larger than expected and vice versa. This is the trend shown by the data points in figure 6b. Participants underestimated the TTC when balls of a larger size were present and vice versa (size-main effect: *F*_2, 20_ = 25.268, *p* < 0.001, $\eta^{2}$ = 0.005). Note that none of the heuristic strategies make different predictions for different sizes with respect to the final response time.

Gravitation and ball size were not the only potential factors influencing the estimates of the remaining TTC. Since the accuracy of the estimates relies on the timing of the observer positions, and they did not proceed to the final landing position (see figure 3a), the final estimates of TTC may not be perfectly accurate. This inaccuracy depends on whether the ball falls in front of or behind the observer’s line of sight ([15] and inset of figure 6c). We can exploit this fact to see if the observed TTC estimates for the different trajectories are consistent with the predicted biases shown in the inset. Figure 6c shows that this is certainly the case with the pattern of errors found in our results (initial angle main effect: *F*_4, 40_ = 7.239, *p* < 0.001, $\eta^{2}$=0.012). Note that the direction of the biases in the TTC estimates is predicted by the final available information from the model depending on the actual position of the actor. This provides strong evidence for the use of the proposed model. This additional evidence, combined with previously reported findings such as heading and kinematics, establishes our model as the most comprehensive explanation for the observed data patterns.

The average temporal errors (approx. 50 ms delayed) and the final errors in locomotion can be attributed to the absence of final feedback, which prevented our participants from correcting their errors. The average final error represents about 14% of the final remaining TTC assuming participants updated their final estimates at the very last possible moment (mean of 350 s before contact).

## 4. Discussion

Dominant theories explaining how humans catch parabolic balls rely on error-nulling heuristics [6,7] in which outfielder movements are coupled to optic variables related to the ball’s motion. While this coupling always predicts successful catches, it can be challenging to identify the specific control variables [9] unless perturbed, non-parabolic trajectories are shown [8]. We propose a model that combines optic variables and two physical constants that are assumed to be known to the participant, gravity and object size, which play no explicit role in previous heuristic strategies.

Our model predicts systematic errors when gravitation or ball size differs from observer expectations. By manipulating these two factors, we assessed whether participant behaviour aligned with our model’s predictions or with heuristics across locomotion and timing judgment phases. Converging evidence from locomotion, kinematics and timing supports our model. Participants altered their paths based on gravitational conditions encountered. This indicates a potential misestimation of TTC which was consistent with combining Earth gravity with the simulated acceleration. For instance, under higher simulated gravities, we observed movements away from the ball. These findings are, contrary to heuristic expectations of forward movement owing to reduced elevation angle (γ) away from the ball, consistent with an overestimated TTC as predicted by our model. Qualitatively distinct predictions from the heuristic strategy remain unchanged even when introducing more free parameters into the heuristic controller. Consequently, it becomes challenging for various heuristic strategies to account for the observed trajectories given the exposed values of optic variables. The previous study by Fink and colleagues [8] contrasts the GOAC and LOT models in ball-catching scenarios, finding evidence for the GOAC model. Although this might appear contradictory to our main findings, it is important to note that under conditions without gravity manipulation, such as in Fink’s study, our model’s predictions closely align with GOAC, to the point of being nearly indistinguishable. The distinction arises only when manipulating gravity, which is key to illustrate the relevance of this environmental constant.

The Bayesian approach in vision science provides distinct perspectives on how priors might be used within the visual system [27]. One perspective would favour that the gravity prior is internally represented as a probability distribution [20,28–30], actively engaged in a Bayesian inference process. Alternatively, the prior can be understood as a tool that describes constraints on scene structure or the environment, thereby specifying the theoretical limits of visual performance. The latter interpretation aligns with Block’s view [31], where Bayesian frameworks are instrumental at the computational level of analysis [32] and define performance limits or specify information content [33] without implying that Bayesian inference processes are represented within the visual system. Although our model, which incorporates Earth’s gravitational acceleration as a parameter, is somewhat eclectic in relation to these views, we lean towards this second interpretation. Our model allows for the possibility that a combination of optical variables could cue the simulated acceleration without relying on internally represented priors.

Unlike gravity, size manipulation only had a marginal effect on the trajectories. This result is consistent with participants giving very little weight to a prior size or accessing the correct size of the ball while it was visible. This can be explained in different ways: by employing available binocular information [34,35], by (correctly) assuming a constant initial distance [13,36,37], or using a combination of both sources of information. However, like gravity, prior size owing to familiarity [38,39] did have an effect on the temporal response (second stage), which was performed after occlusion, which is consistent with prior information becoming more relevant when sensory evidence is absent [40]. Prior known size would become more relevant in the second stage (e.g. judging the remaining TTC or adjusting the final catch) than for guiding locomotion, which is consistent with using size as a metric to estimate passing distance [41]. This explanation applies to both, a size and gravity prior leading to a higher reliance in prior knowledge (figure 6).

One limitation of our model is the exclusion of complex dynamic factors, such as air drag and the Magnus effect. Air drag, which varies with ball size, would influence trajectory and flight time under real-world conditions [14,30]. In such cases, our model’s estimates may be less accurate, potentially leading to TTC errors of up to 10%. However, these errors would diminish significantly within 500 ms before contact, reducing TTC errors to under 50 ms. In our experiment, air drag was not simulated, and the fact that participants’ paths did not differ between ball sizes suggests that observers did not account for air drag when predicting the ball’s trajectory. Further research is needed to investigate this in more detail and to consider how air drag might be incorporated as an additional environmental constraint.

Considering that our model yields accurate initial estimates of landing positions in our experiment, one could think that the ideal path to the interception point would be a straight one to minimize changes in direction. However, our experimental results show that observers consistently follow a slightly curved path towards the interception location, which is in agreement with previous studies [6,8,42]. Our kinematic analysis (figure 5a,b) shows that actors initially favour lateral movement, resulting in slightly curved paths, before moving in depth. This behaviour may result from differing levels of uncertainty and displacement costs associated with lateral versus depth estimates (compare figure 4b and c). For example, the effects of air drag are easier to manage visually in the horizontal plane than in the depth dimension. Similarly, lateral perceptual estimates (e.g. $x_{T}$) tend to be less noisy than in-depth ones, which require more perceptual evidence owing to noisier optic variables. Therefore, our modelled agent independently adjusts its velocity in each dimension based on these running estimates, effectively capturing the observed kinematics.

Our consideration of uncertainty is consistent with the computational approach of Belousov *et al*. [43], which employs an optimal control framework to model heuristics. In their model, an agent uses noisy observations to guide movement, switching between online and predictive control depending on factors such as observation uncertainty and flight duration. For instance, with limited observation time, the agent adopts predictive strategies. A key distinction in our model is its use of optic variables, combined with environmental constants like gravity, to predict trajectories, as opposed to Belousov *et al*., who model states in Cartesian coordinates and do not address how metric positions are derived from optical projections. Our model, however, recovers metric information—such as egocentric landing positions—directly from optical variables by incorporating physical constants like gravity and object size. Importantly, this does not necessarily require assuming the use of metric information, as it can also be body-scaled in a manner consistent with affordances [44]. This approach resonates with studies grounded in the direct perception tradition [45], which emphasize the extraction of three-dimensional layout information from optic variables. Our model thus bridges the gap between interception performance and broader theories of visual perception, elucidating how three-dimensional structure can be inferred from retinal motion under rigidity assumptions [2,46].

## 5. Conclusion

Our model provides a comprehensive framework that bridges the gap between existing interception models and the integration of environmental constants like gravity and size into a perceptual-motor model. This approach enhances our general understanding of interception behaviour and its adaptability to varying environmental conditions. The adaptability demonstrated by participants in response to altered gravitational accelerations illustrates the model’s potential to predict behaviour with different environmental conditions.

## Data Availability

The data, R code [47] for analysis, simulations and figure reproduction, along with an interactive R Shiny application for model exploration, are available at this OSF project: [48]. Supplementary material is available online [49].
